# Supplementary material for: E-Cigarette Aerosol Condensate Leads to Impaired Coronary Endothelial Cell Health and Restricted Angiogenesis
Source: Int J Mol Sci. 2023 Mar 28;24(7):6378. doi: 10.3390/ijms24076378 (PMC10094580; doi:10.3390/ijms24076378)
Supplement: Supplementary file 1 [file ijms-24-06378-s001.zip › ijms-2147745-supplementary.pdf]

Supplementary Figure

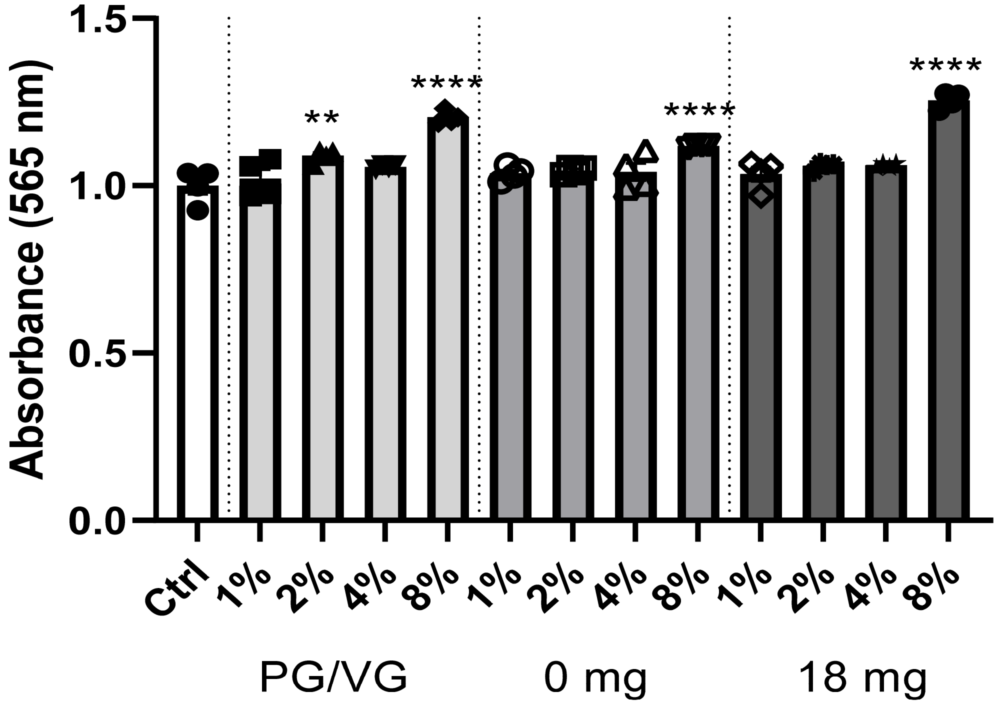

Figure S1. Absorbance of cell-free Endothelial Cell Growth Medium containing different concentrations of EAC after 3 hours of incubation with MTT reagent (10 µL of 5 mg/mL MTT). Results are expressed as mean ± SEM (n=4). One-way ANOVA with Bonferroni post-tests was used for statistical analysis; ***p<0.01*, *****p<0.0001* versus Ctrl.
